# Nursing workload, nurse staffing methodologies and tools: A systematic scoping review and discussion

**DOI:** 10.1016/j.ijnurstu.2019.103487

**Published:** 2020-03

**Authors:** Peter Griffiths, Christina Saville, Jane Ball, Jeremy Jones, Natalie Pattison, Thomas Monks, Safer Nursing Care Study Group

**Affiliations:** a University of Southampton, Health Sciences, United Kingdom; b National Institute for Health Research Applied Research Collaboration (Wessex), United Kingdom; c Department of Learning, Informatics, Management and Ethics, Karolinska Institutet, Sweden; d University of Hertfordshire, School of Health and Social Work, United Kingdom; e East & North Hertfordshire NHS Trust, United Kingdom; f University of Exeter, College of Medicine and Health, United Kingdom

**Keywords:** Patient Classification Systems, Nurse staffing, Nursing workload, Hospital administration, Workforce planning, Personnel Staffing and Scheduling, Nursing administration research, Operations research, Patient safety, Quality of health care, Validation studies, Workload, Costs and cost analysis, Health care economics and organisations, Hospital information systems, Nursing Staff, Hospital

## Abstract

**Background:**

The importance of nurse staffing levels in acute hospital wards is widely recognised but evidence for tools to determine staffing requirements although extensive, has been reported to be weak. Building on a review of reviews undertaken in 2014, we set out to give an overview of the major approaches to assessing nurse staffing requirements and identify recent evidence in order to address unanswered questions including the accuracy and effectiveness of tools.

**Methods:**

We undertook a systematic scoping review. Searches of Medline, the Cochrane Library and CINAHL were used to identify recent primary research, which was reviewed in the context of conclusions from existing reviews.

**Results:**

The published literature is extensive and describes a variety of uses for tools including establishment setting, daily deployment and retrospective review. There are a variety of approaches including professional judgement, simple volume-based methods (such as patient-to-nurse ratios), patient prototype/classification and timed-task approaches. Tools generally attempt to match staffing to a mean average demand or time requirement despite evidence of skewed demand distributions. The largest group of recent studies reported the evaluation of (mainly new) tools and systems, but provides little evidence of impacts on patient care and none on costs. Benefits of staffing levels set using the tools appear to be linked to increased staffing with no evidence of tools providing a more efficient or effective use of a given staff resource. Although there is evidence that staffing assessments made using tools may correlate with other assessments, different systems lead to dramatically different estimates of staffing requirements. While it is evident that there are many sources of variation in demand, the extent to which systems can deliver staffing levels to meet such demand is unclear. The assumption that staffing to meet average need is the optimal response to varying demand is untested and may be incorrect.

**Conclusions:**

Despite the importance of the question and the large volume of publication evidence about nurse staffing methods remains highly limited. There is no evidence to support the choice of any particular tool. Future research should focus on learning more about the use of existing tools rather than simply developing new ones. Priority research questions include how best to use tools to identify the required staffing level to meet varying patient need and the costs and consequences of using tools.

**Tweetable abstract:**

Decades of research on tools to determine nurse staffing requirements is largely uninformative. Little is known about the costs or consequences of widely used tools.

## What is already known about the topic?

• There are many studies showing adverse effects of low nurse staffing on patient outcomes.
• There has been a longstanding interest in developing systems to determine the required staffing level.
• Despite decades of research and a large number of tools, previous reviews have highlighted limited evidence about their use.

## What this paper adds

• Recent years continue to see reports of new staffing tools and systems.
• Important sources of variability are neglected in published reports.
• Benefits are associated with increased staffing levels but the costs and benefits of using a tool, as opposed to simply increasing staffing, remain unknown.

## 1 Introduction

Multiple reviews of research have established that higher registered nurse staffing levels in hospitals are associated with better patient outcomes and improved care quality, including lower risks of in-hospital mortality, shorter lengths of stay and fewer omissions of necessary care (e.g. Brennan et al., 2013; Griffiths et al., 2016, 2018b; Kane et al., 2007; Shekelle, 2013). However, beyond providing an injunction to invest in ‘more’ staff, such studies rarely indicate directly how many staff are required. The ability to determine the ‘right’ number of staff, both to employ and to deploy on any given shift, is an imperative from the perspective of both quality and efficiency of care (Saville et al., 2019). In this paper, we consider the evidence base for approaches to measuring nursing workload and tools used to determine the number of nurses that are required for general acute-care hospital wards.

### 1.1 Nurse staffing levels and outcomes

Low nurse staffing is associated with omissions of essential nursing care (Griffiths et al., 2018b), identified as a key mechanism leading to adverse patient outcomes (Recio-Saucedo et al., 2018). Building on the extensive evidence from cross-sectional studies, recent studies have shown associations at a patient- rather than hospital- or unit-level (Griffiths et al., 2018a, 2019; Needleman et al., 2011b). These include studies involving direct observation of care delivery (Bridges et al., 2019) and studies showing that omissions in care mediate associations between staffing levels and outcomes (Ball et al., 2018; Bruyneel et al., 2015; Griffiths et al., 2018a). While cause and effect cannot be directly inferred from observational studies, the case for a conclusion that low nurse staffing causes harm to patients is increasingly compelling. Perhaps the case is best made by considering the alternative proposition. It seems highly unlikely that there are no adverse outcomes caused by low nurse staffing levels.

Partly as a response to this evidence, policies of mandatory staffing minimums have been much discussed and implemented in a number of jurisdictions, most notably California, USA (Donaldson and Shapiro, 2010; Mark et al., 2013; Royal College of Nursing, 2012). Yet, even where mandatory staffing policies are implemented, patient care needs that cannot be met by the minimum must be identified, and staffing adjusted accordingly. The question of how best to identify the required nurse staffing level remains unanswered.

### 1.2 Staffing tools and methodologies

Determination of appropriate nurse staffing levels and measurement of workload have been studied since the earliest days of research into nursing (e.g. Lewinski-Corwin, 1922). Over the years, there have been many reviews focussing on methods for determining nurse staffing requirements. All have highlighted major deficits in the evidence. The problem is not a simple lack of published literature. One early review of nurse staffing methodologies, published in 1973, included a bibliography of over 1000 studies (Aydelotte, 1973). However, finding no evidence concerning the relative costs or effectiveness of different staffing methods and little evidence for validity or reliability, the authors concluded *“Although the intent of the methodologies is admirable, all are weak”* (p. 57) (Aydelotte, 1973).

Subsequent reviews have had to embrace an ever-growing body of research and an increasing number of systems. A review undertaken for the then Department of Health and Social Services (DHSS) in the UK in 1982 identified over 400 different systems for determining staffing requirements (DHSS Operational Research Service, 1982). Despite the volume of writing, evidence to judge the merits of these systems has remained elusive. Writing in 1994, Edwardson and Giovanetti noted the absence of published scientific evidence for a number of systems, such as GRASP or Medicus, which were in widespread use in North America (Edwardson and Giovannetti, 1994). They also noted that although different systems tended to produce results that were highly correlated, they could nonetheless produce substantially different estimates of the required level of nursing staff for a given patient or unit (Edwardson and Giovannetti, 1994).

Fasoli and Haddock reviewed 63 sources (primary research, theoretical articles and reviews) and again found that there was insufficient evidence for the validity of many current systems for measuring nursing workload and staffing requirements, concluding that systems are not sufficiently accurate for resource allocation or decision-making (Fasoli et al., 2011; Fasoli and Haddock, 2010). Other reviews reinforce this pervasively negative picture of the evidence (Arthur and James, 1994; Butler et al., 2011; Hurst, 2002; Twigg and Duffield, 2009). The field is dominated by descriptive reports of locally developed approaches and none of these reviews found any evidence for the impact of implementation of a tool on outcomes for quality of care, patients or staff (Griffiths et al., 2016).

However, the topic remains important. Identifying low staffing as a significant contributor to *“conditions of appalling care*”, a key recommendation of the Francis Inquiry into the failings of the Mid Staffordshire General Hospital in the United Kingdom was the development of guidance for nurse staffing including:

“…evidence-based tools for establishing what each service is likely to require as a minimum in terms of staff numbers and skill mix.”(p. 1678) (Francis, 2013)

In this paper we aim to give an overview of approaches to measuring nurse staffing requirements for general acute hospital wards, drawing primarily on existing reviews, before presenting a more comprehensive overview of more recent primary research to determine whether (and how) evidence has changed in recent years.

## 2 Review methods and scope

### 2.1 Search strategy and approach to review

The sheer volume of material and unanswered questions identified in other reviews makes this a daunting area to summarise. We describe the current review as systematic in the sense that we aim to be explicit about the approach to identification and selection of literature. However, as we primarily aim to map the literature, identifying recent developments, key features and areas of relative strength and weakness, without necessarily giving each study an in-depth critical appraisal, we consider this a scoping review, serving to summarise findings and identify gaps in the knowledge (Arksey and O'Malley, 2005).

We draw selectively on older authoritative sources and reviews to give a general overview and background to the evidence (including the reviews already cited), using the results of our comprehensive searches and review of reviews undertaken for the National Institute for Health and Care Excellence, NICE (Griffiths et al., 2014) as a key source.

In order to identify more recent studies, we searched Medline, CINAHL (key word only) and The Cochrane Library using the terms “Workload”[key word, MESH] or “Patient Classification”[key word] AND “Personnel Staffing and Scheduling” AND “Nurs*”[key word] or “Nursing”[MESH] and limited results using the OVID Medline sensitive limits for reviews, therapy, clinical prediction guides, costs or economics. We checked the sensitivity of this search, which was designed to be specific, using the results of our earlier more comprehensive search (Griffiths et al., 2014) as a test set. We performed additional searches for citations to existing reviews and for other works by the authors of those reviews (since such reviews might be conducted as a prelude to new empirical research). We also undertook focussed searches on databases for works by key authors and searched the World Wide Web using the names of widely used tools. Searches were completed in mid-December 2018. We looked specifically for new reviews published after 2014 (when searches for our 2014 review of reviews were completed) and primary studies published from 2008 onwards, because the most recent review in our review of reviews was published in 2010 (Fasoli and Haddock, 2010). After removing duplicates, we had 392 recent sources to consider.

### 2.2 Selection of primary research

Consistent with the aims of a scoping review, we took a liberal approach to inclusion for material to review. We included primary studies that described the development, reliability or validity testing of systems/ tools for measuring nursing workload/ predicting staffing requirements; studies that compared the workload as assessed by different measures, or which used a tool as part of a wider study in such a way that it might provide some insight into the validity of tools or another aspect of the determination of nurse staffing requirements; and studies that reported the costs and/or consequences of using a tool, including the impact on patient outcomes. We also included descriptive papers that might not merit the label ‘study’, provided that they included some data. We only included studies that were of direct relevance to staffing on general acute adult inpatient units and so excluded studies focussing exclusively on (for example) intensive or maternity care. However, had we identified material that demonstrated a significant methodological advance or other insight we were open to including it for illustrative purposes.

## 3 Results

### 3.1 Overview of approaches to determining nurse staffing levels

**Fig. 1:**
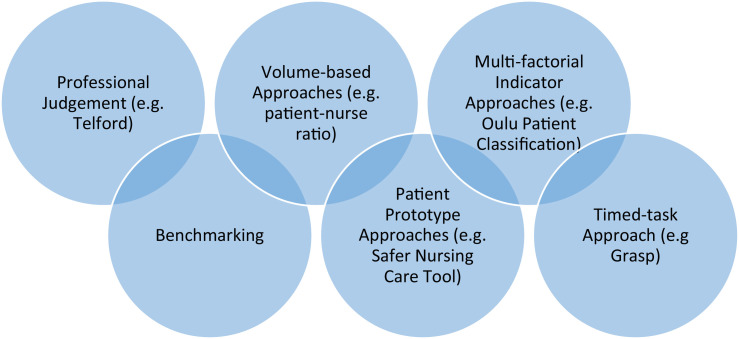
Major approaches for determining nurse staffing requirements.

There are many methods for determining nurse staffing requirements described in the literature. They are generally classified into several broad types (Fig. 1) although the distinction between these approaches is less absolute than it may appear and terminology varies.

Telford's *professional judgement* method (Telford, 1979), first formally described in the UK in the 1970s, provides a way of converting the shift-level staffing plan, decided using expert opinion, into the number of staff to employ. The method describes calculation of the number of nurses to employ (generally referred to in the UK literature as the nursing ‘establishment’) in order to reliably fill the daily staffing plan (planned roster), making allowance for holidays, study leave and sickness/absence. Conversely, this method can be used to infer the daily staffing plan from the whole time equivalent staff employed by a ward, as illustrated by Hurst (2002). The full ‘Telford’ method provides a framework for wider deliberation, but the judgement of required staffing does not require the use of objective measures to determine need (Arthur and James, 1994), hence it is an example of a ‘*professional judgement’-*based approach*.* In recent years, this deliberative approach without formal measurement is reflected in the United States Veteran's Administration staffing methodology (Taylor et al., 2015).

*‘Benchmarking approaches*’ involve using expert judgements to identify suitable comparators, with the staffing levels compared between similar units to establish requirements. For many years this approach was used by the audit commission in the UK (Audit Commission, 2001) to compare nursing establishments and expenditure between units across hospitals. Although characterised by Hurst (2002) as a distinct method, like professional judgement, benchmarking does not involve any formal assessment of patient requirements for nursing care. Rather, consensus methods and expert professional judgement are often used in selecting appropriate benchmarks and so it could be characterised as a particular form of the professional judgement approach, although such characterisation requires that such a judgement is applied. Furthermore, while the process of comparison with similar wards gives the appearance of objectivity, much depends on how the initial staffing levels were arrived at, and there is ample evidence that perceptions of staffing requirements are often anchored to historical staffing levels (Ball et al., 2019; Twigg and Duffield, 2009).

While accounts of professional judgement and benchmarking exercises often focus on determining establishments, both can also be used to determine a daily staffing plan or shift-level *nurse-patient ratio* or equivalent (such as nursing hours per patient). In this way they assign a target number of nursing staff or hours per patient or bed (Hurst, 2002), informing staff deployment decisions. Such approaches specify unit types to which a particular staffing level applies, although categories tend to be broad (e.g. intensive care, general medical surgical and rehabilitation). Some more recent approaches to monitoring workload (see below) extend this approach to take a wider view of activity, for example adding in admissions and discharges over and above the patient census, and therefore we term these patient-nurse ratio approaches *‘volume-based’* approaches*.*

Approaches that appear to set minimum staffing levels per patient, an example of a *volume-based* approach, are sometimes explicit in stating that additional staffing may be required to meet peaks in demand. For example, the legislation that established mandatory nurse-patient ratios in California includes a stipulation that hospitals also use a system for determining individual patient care requirements to identify the need for staffing above the specified minimum (State of California, 1999). Thus, approaches which seek to determine staffing requirements accounting for individual patient variation in need or other factors driving workload can be used as alternatives to, or in conjunction with, minimum staffing levels based purely on patient volumes.

Whereas volume-based approaches measure variation in workload determined by patient counts, other approaches recognise that patients in a given type of ward may have different care requirements. Edwardson and Giovannetti (1994), offer a typology of three main approaches for determining individual patient need: *prototype, task* and *indicator* systems. Hurst also describes three main types: *Patient Classification Systems, timed-task and regression-based* (Hurst et al., 2002).

*Prototype* or *Patient Classification* Systems group patients according to their nursing care needs and assign a required staffing level for each (Fasoli and Haddock, 2010; Hurst, 2002). They use either pre-existing categorisations, e.g. diagnosis-related groups (Fasoli and Haddock, 2010), or bespoke categorisations, e.g. classifications based on levels of acuity and/or dependency groups. The Safer Nursing Care Tool (The Shelford Group, 2014), the most widely used method for determining staffing requirements in England (Ball et al., 2019), is one such system. Patients are allocated to one of five acuity/dependency categories with a weighting (described as a ‘multiplier’) to indicate the required staff to employ associated with patients in each category.

In *task* (or *timed-task*) approaches, a detailed care plan, consisting of specific ‘tasks’, is constructed for each new patient and used to determine the required staffing (Hurst, 2002). Each task is assigned an amount of time. The commercial GRASP system, still widely used in the United States, is an example of such a system (Edwardson and Giovannetti, 1994).

As with prototype approaches, *indicator* approaches ultimately assign patients to categories, in this case based upon ratings across a number of factors that are related to the time required to deliver patient care. These can include broad assessments of condition (e.g. ‘unstable’), states (e.g. ‘non ambulatory’), specific activities (e.g. complex dressings) or needs (e.g. for emotional support or education) (Edwardson and Giovannetti, 1994). The Oulu Patient Classification, part of the RAFAELA system, is one such example. Patients are assigned to one of four classifications, representing different amounts of care required, based upon a weighted rating of care needs across six dimensions (Fagerström and Rainio, 1999). However, the inclusion of some specific activities in Edwardson and Giovennetti's definition of indicator approaches makes it clear that the distinction from task / activity-based systems is not an absolute one. Typically, though, task-based systems take many more elements into account: over 200 in some cases (Edwardson and Giovannetti, 1994).

Hurst also identified *regression*-based approaches, which model the relationship between patient-, ward- and hospital-related variables, and the establishment in adequately-staffed wards (Hurst, 2002). To obtain the recommended establishment for a particular ward, coefficients derived from the regression models are used to estimate the required staffing. There are relatively few examples, although Hoi and colleagues provide one recent example, the Workload Intensity Measurement System (Hoi et al., 2010). In some respects, regression-based models simply represent a particular approach to allocating time across a number of factors within an indicator-based system, rather than directly observing or estimating time linked to specific activities or patient groups. The RAFAELA system, widely used in the Nordic countries, although based on a relatively simple indicator system, uses a regression-based approach to determine the staffing required to deliver an acceptable intensity of nursing work for a given set of patients in a given setting (Fagerström and Rainio, 1999; Fagerstrom and Rauhala, 2007; Rauhala and Fagerström, 2004).

In these more tailored approaches, the method for determining the required times for patient groups or tasks varies. The literature describes the use of both empirical observations and expert opinion to determine the average time associated with tasks or patient classifications (De Cordova et al., 2010; Myny et al., 2014; Myny et al., 2010). In some cases, there is an explicit attempt to make workload/time allocations based on reaching some threshold of quality. For example, wards contributing to the database from which the multipliers for the Safer Nursing Care Tool are derived must meet a predefined standard for care quality (Smith et al., 2009). Non-patient contact time, for example care planning and documentation or other activities that take place away from the bedside (which are not always easily attributable to individual patients), is dealt with in different ways. All approaches consider this, often assigning a fixed percentage time allocation over and above direct care that has been measured.

While some approaches appear to be more precise than others, using detailed patient care plans at one extreme (timed-task) and apparently assuming all patients have similar needs (volume-based) at the other, all use average time allocations, with an unstated assumption that when summed across tasks and patients, individual variation can be accommodated.

#### 3.1.1 Staffing decisions and the use of tools

A number of different decisions can be made using staffing systems and tools, with decisions operating in different time frames (Table 1). Nursing managers must decide in advance how many nursing staff to *employ* (often referred to as the *nursing establishment*) and how many nursing staff to *deploy* each shift, either as a fixed daily staffing plan or in response to immediate demand. Accounts of indicator and task approaches often focus on measuring immediate need (and implicitly deploying staff to meet such need) rather than determining an establishment to fill planned rosters. These are separate but inter-related decisions, which all rely on being able to quantify nursing *workload.* The distinction is sometimes unclear in published accounts and the relationship between these uses tends to be implicit rather than explicit.

**Table 1:** Uses of staffing systems and tools.

Prospective employment	Concurrent deployment	Retrospective review
• Establishment setting: employment and base deployment decisions (long term). • Predict immediate future demand (e.g. next shift)	• Determine current staffing adequacy and guide deployment/redeployment • Prioritise and allocate work to a team	• Review success of staffing plans • Billing and resource use

For example, the Safer Nursing Care Tool was designed to support decisions about the total nursing establishment required on a ward based on meeting the daily needs of a sample of patients (The Shelford Group, 2014). More recently, its core acuity-dependency scoring system has been used to plan and review daily staffing levels, supporting deployment and real-time redeployment decisions, for example using the SafeCare system from the commercial rostering system provider Allocate (Allocate Software, 2017).

There are also examples of tools specifically to balance workload within a unit, which thus focussed primarily on immediate assignments for staff members (Brennan and Daly, 2015; Brennan et al., 2012). Finally, tools can be used retrospectively to review the success of staffing plans (how well the plan met needs) or as a measure of resource use for pricing, budgeting or billing purposes (Kolakowski, 2016).

#### 3.1.2 Overlap between approaches

While the classifications are useful to distinguish broad approaches, the differences are not absolute. For example, professional judgement-based approaches might involve benchmarking to set a fixed establishment for a ward based on an underlying staffing model that aims for a given nurse-patient ratio on each shift and so resembles a volume-based approach. The original determination of the staffing requirement might have involved a detailed appraisal of patient need on a given ward involving many factors similar to those considered in other systems, without a formal calculation of workload based on measurements.

On the other hand, prototype or indicator systems set establishments or daily staffing plans based on a measurement of a sample of individual patient needs, assuming that this can be used to generalise to the patient population as a whole. The establishment, once set, implies that care needs are then met by a fixed nurse-to-patient ratio or number of hours per day, although these ratios may differ between wards. Indeed, a prototype classification system, such as the Safer Nursing Care Tool, resembles a volume-based mandatory minimum staffing policy supplemented by assessment of variation above the base requirement, such as that implemented in California, because there is an implied absolute minimum staffing level per patient, associated with the prototype with the lowest staffing requirement.

#### 3.1.3 Choice of tools

The reviews cited earlier made it clear that there was little basis to prefer any one approach over another based on the available evidence. Professional judgement-based approaches, despite being open to accusations of subjectivity, cannot be readily dismissed without evidence that moving from a judgement-based staffing model to one informed by a tool has improved any outcomes or made more efficient staffing allocations. Existing reviews present no such evidence (Arthur and James, 1994; Aydelotte, 1973; DHSS Operational Research Service, 1982; Fasoli and Haddock, 2010; Griffiths et al., 2016; Hurst, 2002; Twigg and Duffield, 2009). Professional judgement remains central and indeed is incorporated into some tools. One of the most comprehensively researched systems determines the staffing requirement by titration against a subjective report of work intensity (Fagerström and Rainio, 1999; Rauhala and Fagerström, 2004).

The use of subjective judgements would matter little if different approaches gave similar results, but this is not the case. While direct comparisons are relatively rare, it is clear from the available evidence that different systems can give vastly different estimates of required staffing (e.g. Jenkins-Clarke, 1992; O'Brien-Pallas et al., 1991, 1992, 1989). In one study, the five systems tested provided estimates that correlated highly. However, they offered a wide range of average staffing requirements for the same sample of 256 patients, from 6.65 h per patient per day to 11.18 (O'Brien-Pallas et al., 1992).

### 3.2 Recent evidence

From our searches for primary studies we found 37 recent sources to consider. They were diverse in their methods although all were observational studies. We classified the sources according to the main purposes of the articles, although some articles did not clearly sit in a single category and were given a dual classification (see Table 2 for classifications and Table 4 in Supplemental material for fuller descriptions).

**Table 2:** Recent studies/sources used in the review.

Group (number of sources)	Overall description	Sources
Descriptions (9)	Six sources simply described the use of a staffing system but also reported some data, which generally consisted of exemplar graphs or charts of varying workload. Three others provide measures of nursing workload/demand: for different ward designs, for different diagnostic groups and for determining variability in patient need prior to developing a new workload management system.	Fagerström et al. (2014), Fenton and Casey (2015), Gabbay and Bukchin (2009), Hurst (2008, 2009), Kolakowski (2016), Smith et al. (2009), Taylor et al. (2015), The Shelford Group (2014).
Comparisons (4)	These sources compared workload as assessed by different approaches.	Beswick et al. (2010), Hoi et al. (2010), Rivera (2017), Simon et al. (2011)
Tool development (13)	These studies reported on the full or partial development of a new measure or adaptation of an existing measure.	Baernholdt et al. (2010), Brennan et al. (2012), de Cordova et al. (2010), Ferguson-Paré and Bandurchin (2010), Gabbay and Bukchin (2009), Hoi et al. (2010), Hurst et al. (2008), Larson et al. (2017), Morales-Asencio et al. (2015), Myny et al. (2014), Myny et al. (2010), Myny et al. (2012), Perroca (2013)
Evaluation (17)	Sources classified as evaluation included assessments of the reliability or validity of a measure (9 sources); assessment of implementation including usability or user experience of the system (3 sources); and studies that provided some evidence of outcomes or costs of when staffing is guided by a particular method (6 sources).	Brennan and Daly (2015), Brennan et al. (2012), Fagerstrom et al. (2018), Fagerström et al. (2014), Griffiths et al. (2018a), Hurst et al. (2008), Junttila et al. (2016), Larson et al. (2017), Liljamo et al. (2017), Morales-Asencio et al. (2015), Needleman et al. (2011a), Perroca (2013), Smith et al. (2009), Taylor et al. (2015), Twigg et al. (2011), Twigg et al. (2013), van Oostveen et al. (2016)
Operational research (4)	Operational research studies seeking to optimise staffing in the face of varying supply/demand including simulations/mathematical models of different approaches to staff deployment.	Davis et al. (2014), Harper et al. (2010), Kortbeek et al. (2015), Maenhout and Vanhoucke (2013)

#### 3.2.1 Descriptions

These descriptive studies illustrate the currency of a range of approaches including professional judgement (Taylor et al., 2015), prototype (Fenton and Casey, 2015; The Shelford Group, 2014) and indicator systems (Fagerström et al., 2014; Kolakowski, 2016), with at least one explicitly combining approaches (Fagerström et al., 2014). Studies demonstrate variation between wards and from day to day and month to month (e.g. Gabbay and Bukchin, 2009; Smith et al., 2009), arising from the number of patients, the numbers of admissions and discharges, individual patient characteristics and their specific needs (e.g. Fagerström et al., 2014; Hurst, 2009; Smith et al., 2009), as well as contextual factors such as the physical arrangement of the ward (Hurst, 2008).

While demonstrating that measured demand for nursing care can vary considerably, none of the descriptive studies provided a measure that allowed the variation to be directly quantified in terms of variability in the staff required from day to day. Knowledge of this variability would help determine whether a fixed staffing plan is liable to meet patient need on a regular basis. This lack of direct quantification is an important limitation given that tools are used to guide fixed staffing plans.

#### 3.2.2 Comparisons

The findings of earlier studies, showing that different methods can give very different results, are reflected in recent research. Differences between alternative approaches to counting patients for methodologies using hours per patient day appear to be of marginal practical significance (Beswick et al., 2010; Simon et al., 2011), but other factors can make a substantial difference to estimated staffing requirements. Methods that take into account more factors appear to arrive at higher workloads. An unquantified statistically significant increase to workload from including patient turnover in a volume-based measure was noted in one study (Beswick et al., 2010). An acuity- and dependency-based indicator system identified an additional six hours of care per day compared to a standard (fixed) hours per patient day method (Rivera, 2017). A new multifactorial indicator system with additional care categories and revised timings resulted in an estimated nursing requirement that was double that determined by an existing simpler system (Hoi et al., 2010).

#### 3.2.3 Tool development

Many studies (thirteen) report the development of new measures or adaptation of existing measures. Most system types, including professional judgement, volume-based approaches and timed-task feature on this list, adding to the range considered in recent descriptions (above). The measures were often developed for local use only. Typically, papers identify time or some weighting associated with aspects of care or particular groups of patients ‘on average’. However, they generally fail to report or consider variability in the underlying estimates.

That variation around the average time could be important is illustrated in the work of Myny and colleagues in Belgium (Myny et al., 2014, 2010), which as well as being an exception by reporting variability, also represents one of the few examples of a sustained programme of research in recent years. Although the reports were focussed on demonstrating the precision of the mean time estimates they derived, the degree of variation associated with a particular task is well illustrated. The estimated standard time for “partial help with hygienic care in bed” had a 95% confidence interval from 7.6 to 21.2 min. The underlying sample of observations could not be easily determined but the wide confidence intervals appear to result from intrinsic variability rather than simply a small sample. “Settling a bed ridden patient” had an interquartile range from 5 to 25.75 min (Myny et al., 2010).

It may be that prototype approaches, where measures are based on typical care needs of patients fitting a particular profile, are less subject to variation between individuals with the same classification because multiple care needs ‘average out’, but we found no equivalent estimates of variation for such systems. One reason that measures of variability rarely appear may be that despite the external appearance of ‘objectivity’, the times or weights assigned within systems are often wholly or partly arrived at through an expert consensus exercise, for example , Brennan et al. (2012) and Hurst et al. (2008). In part this is likely due to the volume of observation required to obtain reliable time estimates (Myny et al., 2010). It is clear that professional judgement remains an important source of information and validation for any system.

#### 3.2.4 Evaluation

Correlations between measures of staffing requirement or workload have been used to establish validity (e.g. Brennan et al., 2012; Hurst et al., 2008; Larson et al., 2017; Morales-Asencio et al., 2015; Smith et al., 2009). In all but one of these examples, the criterion used to establish validity is, in effect, a professional judgement of demand for nursing care. The centrality of professional judgement as a criterion is demonstrated by the RAFAELA system, in which the Oulu Patient Classification (OPC) weighting that is associated with nurses’ judgements that staffing is ‘optimal’ is used to set target staffing (Fagerström et al., 2014).

Successful implementation of any system requires significant investment to engage and train staff. Taylor and colleagues describe the substantial challenges faced in implementing a professional judgement-based system for the US Veteran's Administration (Taylor et al., 2015). While concluding that their system can be successfully implemented, they highlighted nursing leadership and front line staff buy-in as essential. They also emphasised the importance of staff training and the risk of cynicism if staff invest effort in a new system but see little tangible outcome. Even in the face of broad staff support, a pre-implementation study found that there was insufficient engagement with the measures of staffing adequacy required by the RAFAELA system, and satisfactory reliability also proved hard to achieve (van Oostveen et al., 2016). Nurses can make reliable assessments using a number of systems (Brennan et al., 2012; Liljamo et al., 2017; Perroca, 2013), although achieving inter-rater agreement is not always straightforward and the reliability of ratings in a new setting should not be assumed, even for tools where reliability has been established previously (van Oostveen et al., 2016). Reliability of assessment in “real life” may be considerably lower than that achieved under controlled conditions and there are potential adverse effects on engagement when items that end users consider to be important aspects of care are omitted because of less desirable psychometric properties (Brennan and Daly, 2015).

Given the importance of nurse staffing levels for maintaining the quality of patient care and the significant proportion of hospital budgets spent on staffing wards, there has been remarkably little attention given to the impact of tools or systems. Nonetheless recent years have seen the appearance of some evidence linking a mismatch between staff deployed and a calculated staffing requirement to adverse outcomes. This evidence does not clearly point to any particular measurement system and instead tends to align with evidence showing the benefits of higher staffing levels. These studies give some further indication of the validity of some tools as workload measures, but do not, in general, support conclusions that the tools give ‘optimal’ staffing levels, in the sense of identifying a level at which adverse outcomes are minimised or there are diminishing returns from further increase.

A US study using an unspecified commercial Patient Classification System found that the hazard of death was increased by 2% on every occasion a patient was exposed to a shift with 8 or more hours below the target defined by the system (Needleman et al., 2011a). Mortality was also increased by exposure to shifts with unusually high patient turnover, suggesting that this might be generating additional workload unmeasured by the system.

In Finland, nursing workload above the ‘optimal’ level measured using the OPC was associated with adverse patient outcomes, including increased mortality (Fagerstrom et al., 2018; Junttila et al., 2016). However, nursing workload *below* the optimal level (higher staffing) was associated with improvements in outcomes (Fagerstrom et al., 2018; Junttila et al., 2016), challenging the notion of this staffing level as ‘optimal’. Furthermore, the OPC workload measure was not clearly superior to a simple patient per nurse measure based on analysis of decision curves (Fagerstrom et al., 2018).

More recently, a UK study found that registered nurse staffing below the level planned using the Safer Nursing Care Tool was associated with a 9% increase in the hazard of death in one English hospital trust, although low assistant staffing according to this criterion was not associated with mortality increases (Griffiths et al., 2018a). This study also explored staffing level as a continuous variable and found that the relationship between mortality and registered nurse staffing levels appeared to be linear, with no clear threshold effect at the Safer Nursing Care Tool-recommended level.

After implementing a ‘Nursing Hours per Patient Day’ methodology in three hospitals in Australia, there were increases in staffing levels and improvements in several patient outcomes over time, including mortality (Twigg et al., 2011). This volume-based methodology assigns a minimum staffing level (measured in hours per patient day) for six different ward types, based on the patient case mix and complexity. An accompanying economic analysis estimated the cost per life year gained was AUD$8907 (Twigg et al., 2013).

#### 3.2.5 Operational research

Studies emanating from the tradition of operational research are examples of a larger body of literature that focuses on nurse rostering rather than workload measurement tools (Saville et al., 2019). These studies highlight that rosters based on average staffing requirement may not provide an optimal solution to meet varying patient need.

Two studies determined that optimal staffing in the face of varying patient demand was higher than a level determined by staffing to meet the mean demand (Davis et al., 2014; Harper et al., 2010). In one case, apparent ‘overstaffing’ was associated with net cost savings in modelling, in part because of the potential value of ‘excess’ staff who were available for redeployment to understaffed units (Davis et al., 2014). Other studies modelled the effects of the use of varying configurations of ‘float’ pools to meet fluctuation in demand arising from multiple sources (Kortbeek et al., 2015; Maenhout and Vanhoucke, 2013). These two studies again demonstrate the myriad of sources of variation in demand, and the challenge of matching supply of nursing care to that demand, particularly with an establishment based on the ‘average’ demand, while providing little insight into how demand for nursing care should be measured in the first place.

## 4 Discussion

Writing in 1994, Edwardson and Giovanetti concluded that a number of key questions about nursing workload systems remained unanswered:

• *Do the results of workload measurement systems depart significantly from the professional judgements of practicing nurses?*
• *Does the implementation of a staffing methodology or tool lead to altered staffing levels or, conversely, do historical staffing levels influence the assessment of need?*
• *Do workload measurement systems improve the quality of care?*
• *Do workload measurement systems result in more efficient use of nursing personnel?*

While recent years have seen a continued interest and a significant number of publications, these questions remain largely unanswered. There is evidence that some systems are reliable, that workload measured by a system correlates with other (largely subjective) measures, that low staffing relative to a measured requirement is associated with worse patient outcomes and that increased staffing levels associated with use of a system is associated with improved patient outcomes. However, there is no basis on which to determine that any system gives the ‘correct’ staffing levels.

The results of several workload measurement systems correlate with the professional judgement of practicing nurses, but the correspondence is not perfect and the significance of any discrepancies in estimated staffing requirements is unclear. Despite correlations, different systems can give dramatically different results and so it is clear that there can be no single answer to the questions of whether workload measurement systems result in improvements in the utilisation of nursing personnel*.* The advantage of complex systems over simpler systems is unclear. There is some evidence that the more aspects of care are included in otherwise similar indicator or volume-based systems, the higher the estimated staffing requirement. However, there is little basis on which to judge which is correct other than an evidence base showing higher staffing is associated with better outcomes.

Patient outcomes have been shown to improve when staffing is increased above levels identified as ‘optimal’ using professional judgements and a widely used prototype system. Such a finding is consistent with historical staffing levels and expectations influencing perceptions of what is required. So although professional judgement remains central and no system has been shown to be superior, it too may be systematically biased. Although there are perceptions of benefits from using staffing methodologies, the effect on the costs or quality of care remains unclear and the resources involved in running the systems are unquantified, although the required investments could be considerable (Ball et al., 2019).

Given the significant body of evidence that emphasises the specific association between registered nurse staffing levels or skill mix and outcomes (e.g. Aiken et al., 2017) it is perhaps surprising that the mix of staff is rarely addressed directly in this literature. This may be because many systems have their origins in settings where the contribution of support staff to direct patient care is lower, e.g. the USA (Aiken et al., 2017). The issue of determining skill mix is compounded by the fact that the involvement of support staff in the delivery of nursing care can vary widely (Kessler et al., 2010). Some tools consider only registered or licensed nurses while others, such as the Safer Nursing Care Tool (The Shelford Group, 2014), plan the total nursing team size and defer the skill mix decision to professional judgement.

### 4.1 Sources of variation

The methods described in the literature generally match staffing levels to the average (mean) demand associated with a particular patient group, factor or aspect of care when attempting to estimate current or future staffing requirements. Yet in the face of variable demand, simplistic responses based on the average may not be the best way to use the results of measurement systems. While much of the literature is concerned with measurement and identification of sources of variation, it is poor at quantifying such variation in a way that allows its impact on decision-making to be understood.

When workload distributions are approximately normal with small standard deviations, the mean may be an appropriate basis for planning, as the workload will vary from the mean by a relatively small amount. Assuming some degree of flexibility in the work capacity of a given group of staff, most patients’ needs might be safely accommodated most of the time. While some systems such as RAFAELA are explicit about an acceptable degree of variation from the mean (Fagerström et al., 2014), this is rare, and the impact on safety of small deviations has not been widely researched.

However, both substantial variability and skewed distributions seem more plausible. Reports rarely provide estimates of variation in time required for specific aspects of care, but the few that do show that variation around the mean is considerable (Myny et al., 2014). Left (negatively) skewed ward occupancy distributions have been reported (Davis et al., 2014). When this is the case, mean staffing requirements are lower than the median, leading to relative understaffing more than 50% of the time if the mean is used.

Even where a mean adequately allows staff to meet variable demand, it is often unclear how much care needs to be observed to establish a reliable mean. As is clear from Myny et al. (2010), estimating reliable means can be challenging even in a large scale study. The basis on which recommended observation periods were determined for widely used systems such as the Safer Nursing Care Tool is unclear because variation is not reported.

Variation in demand arises at multiple levels, for example patient census, need per patient and time taken to deliver care for a patient with a given set of needs. While some systems account for these factors to some extent, they rarely consider that the averages they use to determine staffing requirements, associated with a given factor, are also subject to variation. So while a task-based system may recognise that different patients require very different care, in assigning an average time it does not account for the variability in time taken to complete a task. In Table 3, we summarise some major sources of variation. Variation around the average may be compounded as multiple aspects of care are considered, or may tend to ‘average out’, but this is simply unknown.

**Table 3:** Sources of variation in demand for and supply of nursing care.

Demand	Supply
Differing care needs • *Different patients have different need, even within the same prototype* • *Variability unknown*	Staff sickness/absence • *Relatively rare occurrence with non-random clustering and seasonal variation*
Varying time to deliver care • *Different lengths of time to undertake the same aspect of care (may be patient- or staff-related)*	Staff leave (holiday and study) • *Predictable seasonal variation*
Patient census/occupancy • *Variation between and within days, known to be left skewed*	Vacancies • *Unpredictable with non-random clustering*
Patient turnover (admission/discharge) • *Considerable variation between and within wards, potentially left skewed.* Ward layout • *Potentially systematic alteration in time required for some care*	Varying time to deliver care • *Different staff may be more or less efficient at performing care and multi-tasking during care delivery*
	

While task-based systems are challenged by the need to specify and time all aspects of nursing work, prototype systems cannot account for variation associated with activities that are not directly linked to the patient prototype. For example, patient turnover generates substantial nursing work (Myny et al., 2012), which is highly variable between and within wards, with some predictable sources of variation (such as day of the week) (Griffiths et al., 2018a). Such variation is not easy to account for in a patient prototype because patients are admitted or discharged at points in time, while the prototype does not change.

Few systems formally consider non-patient factors that may influence workload. For example, while evidence that ward layout may alter staffing requirements is limited (Hurst, 2008), simple factors influenced by layout such as travel distances and opportunity for patient surveillance are recognised as having the potential to generate considerable variation in workload (Maben et al., 2016, 2015). While variation arising from factors such as layout can be accommodated if times required are estimated for each unit, this does raise a final issue.

Variation is often systematic and just as demand is variable, so is the supply of staff to meet that demand (see Table 3). This is a particular issue when planning establishments and advance rosters to meet need. As an example, in order to ensure that there are sufficient staff available to provide cover on wards, the literature describes the need to add an “uplift” to establishments to allow for staff sickness (Hurst, 2002; Telford, 1979). However, staff sickness does not occur uniformly. Rather it occurs in clusters, with clear seasonal patterns and variation by day of the week (Barham and Begum, 2005). Allowing a small percentage of additional staff based on the average percentage of time lost does not mean that sufficient staff are available to cover days or weeks when staff are actually absent.

### 4.2 ‘Optimal’ staffing

Each staffing method makes an underlying assumption about what constitutes ‘adequate’, ‘safe’ or ‘quality’ staffing, although these are often implicit. The staffing to deliver the ‘right’ frequency and length of nursing tasks in the timed-task approach, and the ‘right’ amount of care per patient in the nurse-patient ratio approach must be decided upon. These parameters are generally obtained from expert judgement, from observations of care provided or from existing establishments, ideally in settings deemed to meet some quality criteria (Hurst, 2002). The question of whether this staffing level is ‘optimal’, or what criteria might define an optimal staffing level is rarely, if ever, addressed.

There is evidence that staffing to the ‘optimal’ level defined by the RAFAELA tool is associated with reduced mortality when compared to lower staffing (Junttila et al., 2016) but since mortality is further reduced by staffing at higher levels, it is hard to conclude that this staffing level is, truly, optimal. It is, in effect, a professional judgement about what constitutes reasonable staffing, which is, in turn, bounded by historical expectations (Taylor et al., 2015; Telford, 1979). While this question arises in relation to the RAFAELA tool, because it explicitly identifies an optimum staffing level, the issue applies to all systems. While tools can motivate staffing increases it is also possible that they could restrict staffing at a level that is not clearly ‘optimal’.

The appropriate response to variation in the productivity of staff, related to factors such as experience or efficient deployment of a team, also makes any definition of an ‘optimal’ staffing level a challenge. While it seems important to recognise that (for example) less experienced staff may be less able to meet a given level of demand and thus require some additional support, setting a lower staffing level based on the relative efficiency of a team may appear to be punishing success. Furthermore, while most systems emphasise measurement of demand, optimal management of staffing involves achieving an appropriate balance between supply and demand. ‘Optimal’ staffing levels may be lower if peaks in demand can be reduced (Litvak et al., 2005; Litvak and Laskowski-Jones, 2011). Nursing services do not operate in isolation and the demand for nursing care and the required level of staff may also change as inputs from other staff groups vary. Perhaps, above all, this illustrates that there is a limit to what can be achieved through measurement, both because of the fallible nature of the measures, but also because of the complex judgements that are required.

### 4.3 Limitations

The volume of literature considered for this review and the wide range of questions addressed means that we have not focussed on critiquing specific studies or attempting to draw conclusions about any particular approach. We may have missed some recent studies or older studies about some of the tools featuring in the more recent research. However, our approach of building on existing reviews and our extensive searches means that it is unlikely that we have missed substantial volumes of research that would lead to an overall different conclusion.

### 4.4 Future research

Staff costs and patient outcomes using different systems have rarely been compared. Controlled trials comparing outcomes of staffing guided by tools with other approaches may be challenging to undertake, but are by no means impossible to conceive. Cluster randomised trials may be feasible and controlled before-and-after studies of staffing systems have been reported or are underway (Drennan et al., 2018). Because there are so many unanswered questions much progress can be made outside a trial framework. Natural variation around target staffing levels (for example due to staff sickness) provide further opportunity to study the association of target staffing levels with outcomes using quasi-experimental methods. Questions that remain unanswered about many tools include the extent to which they truly identify a level of staffing sufficient to meet the needs of a ward of patients, and the number of observations required to get an accurate baseline to estimate average need. The apparently simple assumption, that staffing to meet average need is the optimal response to varying demand, is also untested empirically, although research reviewed here suggests this assumption is likely to be incorrect. For systems designed to determine ward establishments, the extent to which the establishments efficiently or effectively deliver staffing levels to match varying patient need (either with or without additional flexible staffing) can be addressed in observational and simulation studies.

## 5 Conclusions

The volume of literature on staffing methodologies is vast and growing. However, there is no substantial evidence base on which to select any particular method or tool. There has been a repeated pattern whereby new tools are developed with little programmatic research addressed at existing tools, even when they are widely used. The extensive research reporting the development of the RAFAELA system stands out as an honourable exception in this regard, although neither costs nor effects of using the tool compared to another tool or no tool at all have been reported. Benefits associated with tools appear to be based on increased staffing levels.

Despite the lack of evidence, an appetite for formal systems and tools exists. While professional judgement remains the nearest to a gold standard, the desire to use a tool or other formal system to support and indeed justify such a judgement has remained a constant theme that can be traced back to Telford's work in the 1970s in the UK, and no doubt beyond. While limitations in tools have continually motivated the development of new approaches, limited evidence means it is hard to determine if existing approaches may be ‘good enough’ or if new approaches are any better in practice. The lack of discernible progress in building an evidence base leads us to conclude that rather than continue to develop new tools, it is time to take a much closer look at those already in use and to investigate the best way to use them and the costs and the consequences of doing so.

## Conflict of interest

Other than the project funding, the authors declare no competing interests that might be perceived as influencing the results of this paper.

## Appendix Supplementary materials

Image, application 1
