# Supplementary material for: Nursing workload, nurse staffing methodologies and tools: A systematic scoping review and discussion
Source: Int J Nurs Stud. 2020 Mar;103:103487. doi: 10.1016/j.ijnurstu.2019.103487 (PMC7086229; doi:10.1016/j.ijnurstu.2019.103487)
Supplement: Supplementary file 1 [file mmc1.pdf]

Table 4 recent studies included in the review

Source / Reference	type of measure	Single centre?	Main Theme	Key Points / findings
Beswick, S., et al. (2010). "Comparison of nurse workload approaches." Journal of Nursing Management **18**(5): 592-598.	patient volume	Single (2 units)	Comparison	Compared workload measures based on simple HPPD with midnight census vs intra day measures (no significant difference) vs measure including turnover (significantly increased HPPD)
Rivera, K. (2017). A Comparison of a Standardized Method of Identifying Nursing Staff Needs with Measures of Nursing Staff Needs by Patient Acuity. Ann Arbor, The William Paterson University of New Jersey. **10637025:** 58.	prototype / patient volume	Single (?)	Comparison	Compared staffing requirement based on HPPD vs an acuity measure
Simon, M., et al. (2011). "Midnight census revisited: Reliability of patient day measurements in US hospital units." International Journal of Nursing Studies **48**(1): 56-61.	patient volume	Multi (54 Hospitals, 260 Units)	Comparison	Compared different patient count methods used in calcualtion hours per patient day and found that while agreement between methods was high there was some evidence of bias but midnight census did not differ substntially from the 'gold standard' (multiple census approach)
Fagerström, L., et al. (2014). "The RAFAELA system: a workforce planning tool for nurse staffing and human resource management: Lisbeth Fagerström and colleagues describe a method pioneered in Finnish hospitals that aims to uphold staffing levels in accordance with patients' care needs." Nursing Management 21(2): 30-36.	indicator / professional judgement	N/A	Description	Descrtive report which includes some example data from the use of the Rafaela system
Fenton, K. and A. Casey (2015). "A tool to calculate safe nurse staffing levels." Nursing Times **111**(3): 12-14.	prototype	N/A	Description	Descriptive report on the use of the SNCT
Hurst, K. (2008). "UK ward design: Patient dependency, nursing workload, staffing and quality--An observational study." International Journal of Nursing Studies **45**(3): 370-381.	prototype	Multi centre (40 hospital / 375 wards)	Description	Calculated average workload associated with various hospital ward designs using Hurst's acuity / dependency / quality mesaure concluding that some ward designs were assocaited with higher workloads than otehrs
Hurst, K. (2009). Nursing and payment By Results: Understanding the Cost of Care (RCN Policy Briefing: 11/2009). London, Royal College of Nursing.	prototype	Multi centre (10 hospital / 40 wards)	Description	Calculated average nursing costs associated with various Health Resourge Groups (diagnoses) using Hurst's acuity / dependency / quality mesaure
Kolakowski, D. (2016). "Constructing a nursing budget using a patient classification system." Nurs Manage **47**(2): 14-16.	unspecified PCS	N/A	Description	Simple description of setting a budget from a an unspecified patient classification system.
The Shelford group (2014). Safer Nursing Care Tool Implementation Resource Pack, The Shelford Group.	prototype	N/A	Description	Manual describing the use of the SNCT. Includes a borad descrption of the volume of observations that udnerly the care multpliers that have been developed
Smith, S., et al. (2009). "Developing, testing and applying instruments for measuring rising dependency-acuity's impact on ward staffing and quality." International Journal of Health Care Quality Assurance 22(1): 30-39.	prototype	Multi (3 hospitals)	Description & evaluation	Dscribes the use and development of the SNCT (then AUKUH) instrment including comparison with an exisitng dependecy measure (crobach's alpha .99)
Taylor, B., et al. (2015). "Evaluating the Veterans Health Administration's Staffing Methodology Model: A Reliable Approach." Nursing Economics 33(1): 36-40, 66.	professional Judgement	N/A	Description & evaluation	Qualitaitve evalaution of feedback (broadly positive) on a formal professional judgement and becnhmarking based staffing review approach.

Table 4 recent studies included in the review

Source / Reference	type of measure	Single centre?	Main Theme	Key Points / findings
Baernholdt, M., et al. (2010). "Using clinical data to capture nurse workload: implications for staffing and safety." CIN: Computers, Informatics, Nursing **28**(4): 229-234.	patient volume	Single (whole hospital)	Development	Describes a measure based on routine clincial data including patient numbers, turnover (admissions and discharges)
De Cordova, P. B., et al. (2010). "Using the nursing interventions classification as a potential measure of nurse workload." Journal of Nursing Care Quality **25**(1): 39.	timed task	N/A	Development	Consensus exercise on timings associated with NIC domains / interventions
Ferguson-Paré, M. and A. Bandurchin (2010). "The Ontario nursing workload demonstration projects: Rethinking how we measure, cost and plan the work of nurses." Nursing Leadership **23**(Special Issue).	indicator	Multi centre (3 sites / 6 units)	Development	Early stage work determining factors to be considered in a staffing system, including timing studies across 12 broad activity areas
Myny, D., et al. (2010). "Determination of standard times of nursing activities based on a Nursing Minimum Dataset." Journal of Advanced Nursing **66**(1): 92-102.	timed task	Multi (18 hospitals 48 units)	Development	Established standard times for 102 nursing activities based on observations and self report
Myny, D., et al. (2012). "Determining a set of measurable and relevant factors affecting nursing workload in the acute care hospital setting: a cross-sectional study." International Journal of Nursing Studies **49**(4): 427-436.	timed task	N/A	Development	Assesssed a wide number of factors influencing nursing workload (other than acuity) in the context of developing a wokload measure. Most important factors increasing workload are interuptions and patient turnover
Hoi, S. Y., et al. (2010). "Determining nurse staffing needs: the workload intensity measurement system." Journal of Nursing Management **18**(1): 44-53.	indicator	Single (1500 beds)	Development & comparison	Developed an indicator system based on care times assocaited with a range of nursing diagnoses + associated activites & used this to identify staffing requirements compared to those from a previous (unreported) study.
Myny, D., et al. (2014). "Validation of standard times and influencing factors during the development of the Workload Indicator for Nursing." Journal of Advanced Nursing **70**(3): 674-686.	timed task	Multi (4 hospitals 23 units)	Development & evalaution	Reports cross validation of a measure based on a NMD with nurses direct recording of care time. Found high correlations but significant differences between time as estiamted from the two measures
Perroca, M. G. (2013). "The new version of a patient classification instrument: assessment of psychometric properties." Journal of Advanced Nursing **69**(8): 1862-1868.	indicator	Single (10 Units)	Development & evalaution	Assessed inter-rater reliability and factor structure of a multi-item PCI . Weighted Kappa showed strong agreement
Brennan, C. W., et al. (2012). "The oncology acuity tool: a reliable, valid method for measuring patient acuity for nurse assignment decisions." Journal of Nursing Measurement 20(3): 155.	indicator / professional judgement	Single (1 Unit)	Development & evaluation	Describes the development of a multi-factor acuity measure + content validation, reliability testing and validation by measures of assocaition with outcome and a direct nurse assessment (expert judgement) of acuity

Table 4 recent studies included in the review

Source / Reference	type of measure	Single centre?	Main Theme	Key Points / findings
Hurst, K., et al. (2008). "Calculating staffing requirements." Nursing Management 15(4): 26-34.	prototype / professional judgement	Multi-center (3 hospital 16 'care groups')	Development & evaluation	Describes development and validation of the AUKUH multipliers (determined by profesional judgement) by rating against an exisiting categorisation scheme with underlying observations of time to delvier care on high quality wards
Larson, E. L., et al. (2017). "Assessing Intensity of Nursing Care Needs Using Electronically Available Data." Comput Inform Nurs 35(12): 617-623.	indicator / professional judgement	Single (whole hospital)	Development & evaluation	Development of a sytem drawing on data from ekectonic systems including procedures to develop an intensity index, used professional judgement of nursing care intensity as the criterion measure (showing high correlation)
Morales-Asencio, J. M., et al. (2015). "Design and validation of the INICIARE instrument, for the assessment of dependency level in acutely ill hospitalised patients." <u>Journal of Clinical Nursing</u> **24**(5-6): 761-777.	indicator	Multi (2 hospitals)	Development & evaluation	Reports the development of an Instrument based on NOC with reports of inter item correlation and validation by corrrealtion with the barthel Index.
Gabbay, U. and M. Bukchin (2009). "Does daily nurse staffing match ward workload variability? Three hospitals' experiences." International Journal of Health Care Quality Assurance 22(6): 625-641.	patient volume	Multi centre (3 sites / 40 units)	Development / descriptive study	Tracked worforice / workload using simple nurse to patient measures to demonstrate variability in demand and capacity. Intended as preliminay to developing worload management systems
Brennan, C. W. and B. J. Daly (2015). "Methodological challenges of validating a clinical decision-making tool in the practice environment." Western Journal of Nursing Research 37(4): 536-545.	indicator	Single (1 Unit)	Evaluation	Reports on 'methodological challenges' encoutnered in developing a new tool but content is largely based on perspectives of potential end uses on aspects of the tool including necessary content.
Fagerstrom, L., et al. (2018). "Nursing workload, patient safety incidents and mortality: an observational study from Finland." BMJ Open 8(4): e016367.	indicator / patient volume / profesional judgement	Multi -centre (4 hospitals / 36 units)	Evaluation	Looked at assocaition beteween OPC per nurse (Rafaela system) and safety incidents. Showed odds of patient safet incident was increased when staffing was below optimal. However it was decreased by staffin above optimal however effect sizes and model fit were only marginally better than patient/nurse ratios
Twigg, et.al., 2013. The economic benefits of increased levels of nursing care in the hospital setting. J Adv Nurs 69 (10), 2253-2261.	patient volume	Multi (3 hospitals)	Evaluation	Befre and after study of implementation of NHPPD method to determine nurse staffing in W. Australian Hospitals. Implementation of staffing according to the system was assocaited with an increase in staff and judged to be cost effective (low cost per life saved) see Twigg et al 2011
Twigg, D., et al. (2011). "The impact of the nursing hours per patient day (NHPPD) staffing method on patient outcomes: a retrospective analysis of patient and staffing data." International Journal of Nursing Studies 48(5): 540-548	patient volume	Multi (3 hospitals)	Evaluation	Befre and after study of implementation of NHPPD method to determine nurse staffing in W. Australian Hospitals. Implementation of staffing according to the system was assocaited with an increase in staff and reduced mortality. ee Twigg et al 2011
van Oostveen, C. J., et al. (2016). "Pre-implementation studies of a workforce planning tool for nurse staffing and human resource management in university hospitals." <u>Journal of Nursing Management</u> **24**(2): 184-191.	indicator / professional judgement	Multi (2 hospitals 12 wards)	Evaluation	Implementation study of the Rafaela system found low agreemenet (absolute) on OPCS measures in some settings and poor completion of the PAONCIL instrument required to determine optimal intensity. Nurses evaluations were mixed
Griffiths, P., Ball, J., Bloor, K., Böhning, D., Briggs, J., Dall'Ora, C., Iongh, A.D., Jones, J., Kovacs, C., Maruotti, A., Meredith, P., Prytherch, D., Saucedo, A.R., Redfern, O., Schmidt, P., Sinden, N., Smith, G., 2018. Nurse staffing levels, missed vital signs and mortality in hospitals: retrospective longitudinal observational study. Health Services and Delivery Research Journal 6, 38.	prototype	Single (32 units)	Evaluation	When RN staffing fell below the planned level, determined through periodic review using the SNCT (prototype system) mortality was increased. High patient turmover relative to unit mean was also assocaited with significantly increased mortality

Table 4 recent studies included in the review

Source / Reference	type of measure	Single centre?	Main Theme	Key Points / findings
Junttila, J. K., et al. (2016). "Hospital mortality and optimality of nursing workload: A study on the predictive validity of the RAFAELA Nursing Intensity and Staffing system." International Journal of Nursing Studies **60**: 46-53.	indicator / professional judgement	Multi centre (2 hospital / 34 units)	Evaluation	Looked at assocaition beteween OPC per nurse (Rafaela system) and mortality. Showed odds death were increased when staffing was below optimal. However it was decreased by staffing above optimal
Liljamo, P., et al. (2017). "Quality of nursing intensity data: inter-rater reliability of the patient classification after two decades in clinical use." Journal of Advanced Nursing 73(9): 2248-2259.	indicator	Single (32 units)	Evaluation	Inter-rater relaibility using OPC (Rafaela syatem) showing moderate agreement on overal OPC category although weighted kappa shows strong areement. Some sub scales show fair agreement only but again generally weighted kappas indicate substantial agreement
Needleman, J., et al. (2011). "Nurse staffing and inpatient hospital mortality." New England Journal of Medicine **364**(11): 1037-1045.	unspecified PCS	Single (43 Units)	Evaluation	When staffing fell below the target set by a PCS mortalitty was increased. High patient turmover relative to unit mean was also assocaited with significantly increased mortality
Davis, A., et al. (2014). "Nurse Staffing under Demand Uncertainty to Reduce Costs and Enhance Patient Safety." Asia-Pacific Journal of Operational Research 31(01): 1450005.	patient volume	Multi (2 units / 2 hospitals)	Operational Research Model	Calculated optimal staffing in the face of variable demand (patient census based only) using a newsvendor model and calculated cost savings (relative to mean) associated with overstaffing according to the model for general.
Harper, P. R., et al. (2017). "Modelling the size and skill-mix of hospital nursing teams." Journal of the Operational Research Society 61(5): 768-779.	prototype/ patient volume	Single (6 units)	Operational Research Model	Calculated optimal staffing in the face of variable demand (patient census and acuity / dependency based measures "informed" by Hurst's work) and estimated required WTE equivelent staff as higher, compared to current (average) based approaches
Kortbeek, N., et al. (2015). "Flexible nurse staffing based on hourly bed census predictions." International Journal of Production Economics 161(167–180): 167-180.	patient volume	Single (4 units)	Operational Research Model	Compares staffing required based on a predicitive tool based on patient census compared to a full (nurses to bed) model & finds that census based models require fewer staff with mixed findings on the use of a flexible staffing pool
Maenhout, B. and M. Vanhoucke (2013). "An integrated nurse staffing and scheduling analysis for longer-term nursing staff allocation problems." Omega **41**(2): 485-499.	patient volume	N/A	Operational Research Model	Models different approaches to staffing with varing compositions of ft/ pt / float unit staff based on an unclear workload / demand model, presumed to be a fixed nurse to bed measure. Variations considered appear to be primarily supply
